# Stoichiometry-Selective Antagonism of α4β2 Nicotinic Acetylcholine Receptors by Fluoroquinolone Antibiotics

**DOI:** 10.1021/acschemneuro.2c00200

**Published:** 2022-06-03

**Authors:** Victoria R. Sanders, Aaron Sweeney, Maya Topf, Neil S. Millar

**Affiliations:** † Division of Biosciences, University College London, London WC1E 6BT, United Kingdom; ‡ Institute of Structural and Molecular Biology, Birkbeck College, London WC1E 7HX, United Kingdom

**Keywords:** Nicotinic acetylcholine receptor, subunit stoichiometry, antagonist, quinolone, antibiotic, pefloxacin

## Abstract

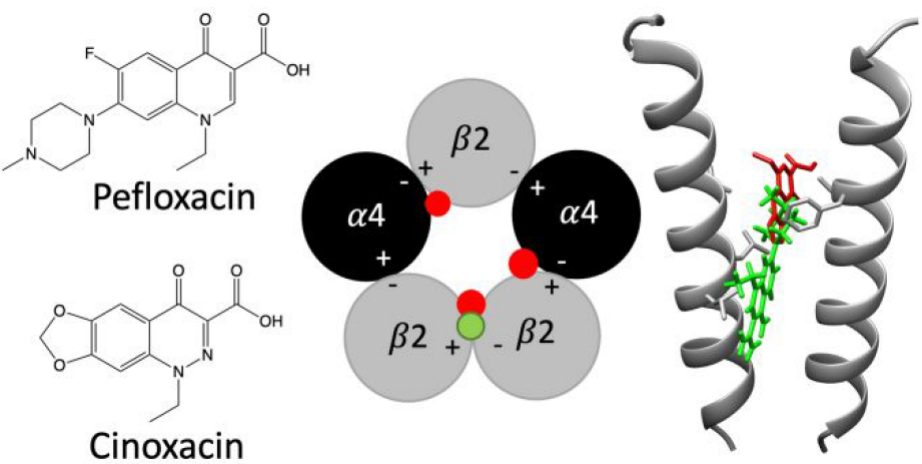

Quinolone antibiotics disrupt bacterial DNA synthesis by interacting with DNA gyrase and topoisomerase IV. However, in addition, they have been shown to act as inhibitors of pentameric ligand-gated ion channels such as GABA_A_ receptors and the α7 nicotinic acetylcholine receptor (nAChR). In the present study, we have examined the effects of quinolone antibiotics on the human α4β2 nAChR, an important subtype that is widely expressed in the central nervous system. A key feature of α4β2 nAChRs is their ability to coassemble into two distinct stoichiometries, (α4)_2_(β2)_3_ and (α4)_3_(β2)_2_, which results in differing affinities for acetylcholine. The effects of nine quinolone antibiotics were examined on both stoichiometries of the α4β2 receptor by two-electrode voltage-clamp recording. All compounds exhibited significant inhibition of α4β2 nAChRs. However, all of the fluoroquinolone antibiotics examined (ciprofloxacin, enoxacin, enrofloxacin, difloxacin, norfloxacin, pefloxacin, and sparfloxacin) were significantly more potent inhibitors of (α4)_2_(β2)_3_ nAChRs than of (α4)_3_(β2)_2_ nAChRs. This stoichiometry-selective effect was most pronounced with pefloxacin, which inhibited (α4)_2_(β2)_3_ nAChRs with an IC_50_ of 26.4 ± 3.4 μM but displayed no significant inhibition of (α4)_3_(β2)_2_ nAChRs. In contrast, two nonfluorinated quinolone antibiotics (cinoxacin and oxolinic acid) exhibited no selectivity in their inhibition of the two stoichiometries of α4β2. Computational docking studies suggest that pefloxacin interacts selectively with an allosteric transmembrane site at the β2(+)/β2(−) subunit interface, which is consistent with its selective inhibition of (α4)_2_(β2)_3_. These findings concerning the antagonist effects of fluoroquinolones provide further evidence that differences in the subunit stoichiometry of heteromeric nAChRs can result in substantial differences in pharmacological properties.

## Introduction

Nicotinic acetylcholine receptors (nAChRs) form part of the superfamily of pentameric ligand-gated ion channels, which includes receptors for 5-hydroxytryptamine (5-HT), γ-aminobutyric acid (GABA), and glycine.^1^ Seventeen nAChR subunits have been identified in vertebrates (α1−α10, β1−β4, γ, δ, and ε) that can coassemble in a variety of combinations to generate a diverse family of pharmacologically distinct nAChR subtypes, including both heteromeric subunit combinations (such as α4β2) and homomeric complexes (such as α7).^2^ Further complexity can arise as a consequence of nAChR subunits coassembling with different stoichiometries. For example, the α4 and β2 subunits can coassemble into pentameric complexes containing either two α4 and three β2 subunits ((α4)_2_(β2)_3_) or three α4 and two β2 subunits ((α4)_2_(β2)_3_).^3^ As has been reported previously, the two stoichiometries of α4β2 nAChR differ in their sensitivity to acetylcholine (ACh) and, as a consequence, are often referred to as “high-sensitivity” and “low-sensitivity” subtypes, respectively.^4^ Receptors containing α4 and β2 subunits mediate the effects of nicotine associated with tobacco smoking and are the site of action of drugs used to assist with smoking cessation.^5^ In addition, α4β2 nAChRs are targets for drug discovery in areas such as cognition, attention, and pain.^6−8^ In recent years, considerable attention has focused on studies of allosteric modulators of nAChRs that are thought to bind within the receptor’s transmembrane domain.^9,10^

**Figure 1:**
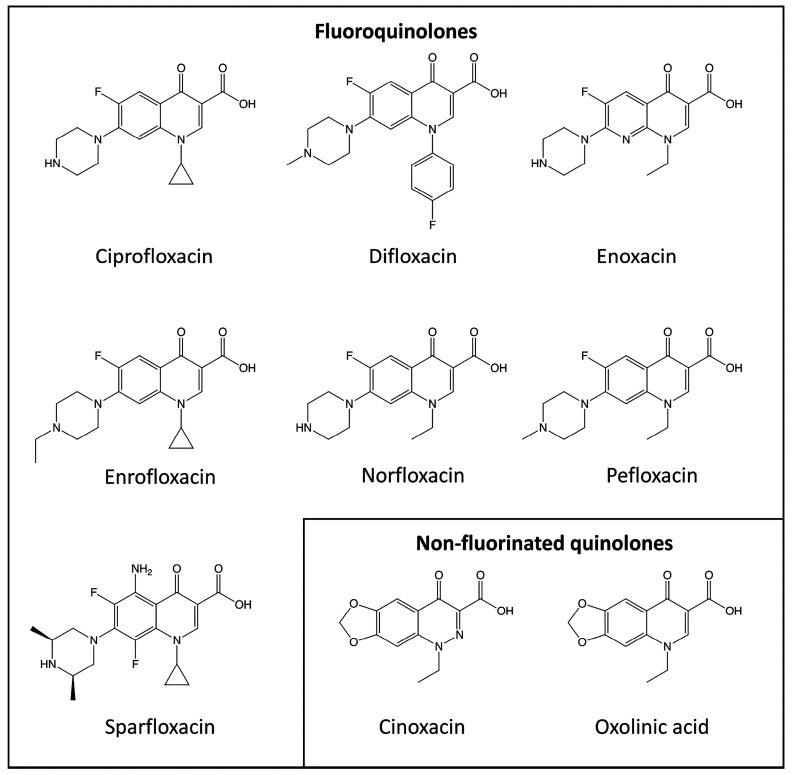
Chemical structures of quinolone antibiotics. The effects of seven fluoroquinolone antibiotics (ciprofloxacin, difloxacin, enoxacin, enrofloxacin, norfloxacin, pefloxacin, and sparfloxacin) and two nonfluorinated quinolone antibiotics (cinoxacin and oxolinic acid) were examined in the present study.

Quinolone antibiotics interact with two distinct targets within bacterial cells, DNA gyrase (DNAG) and topoisomerase IV, both of which are involved in bacterial DNA synthesis.^11^ Quinolones inhibit DNA synthesis by stabilizing complexes of DNA and topoisomerase IV or DNAG which blocks the progression of the replication fork.^11^ However, previous studies have indicated that quinolone antibiotics can also modulate pentameric neurotransmitter-gated ion channels. For example, they have been reported to inhibit ionotropic receptors for GABA (GABA_A_ receptors)^12−15^ and also human α7 nAChRs.^16^ In the case of α7 nAChRs, pefloxacin was identified as a potential allosteric modulator (interacting with the α7 nAChR transmembrane domain) on the basis of virtual screening,^16^ performed with a revised homology model of the α7 nAChR,^17^ and was subsequently shown to act as a noncompetitive antagonist on α7 nAChRs.^16^ Here, we have examined the effects of a series of nine quinolone antibiotics (Figure 1), including pefloxacin, on the two stoichiometries of the human α4β2 nAChR by two-electrode voltage-clamp recording of cloned receptor subunits expressed in *Xenopus* oocytes.

## Materials and Methods

### Plasmids and Reagents

Ciprofloxacin, enrofloxacin, difloxacin, and sparfloxacin were purchased from Sigma-Aldrich (Gillingham, U.K.). Pefloxacin, cinoxacin, and oxolinic acid were purchased from Santa-Cruz Biotechnology (Dallas, TX, USA). Enoxacin was purchased from TOKU-E (Washington, USA), and norfloxacin was purchased from Merck Life Science UK Ltd. (Southampton, U.K.). Stock solutions of antibiotics (100 mM) were prepared in DMSO, with the exception of enoxacin which was prepared in 1 M NaOH. Stock solutions were stored at −20 °C before use.

### Plasmids and Site-Directed Mutagenesis

Human nAChR subunit cDNAs in plasmid expression vector pSP64GL (pSP64GL-α4 and pSP64GL-β2) have been described previously.^18^ Site-directed mutagenesis (to generate plasmids pSP64GL-α4^L283A^, pSP64GL-α4^S284A^, and pSP64GL-β2^V278A^) was performed using the QuikChange mutagenesis kit (Agilent Technologies) and verified by nucleotide sequencing (Source Bioscience). Note that the numbering of these amino acids in the human nAChR α4 and β2 subunits is based on the intact protein sequence (including the signal sequence), as indicated in the EMBL/GenBank database entries NP_000735.1 and NP_000739.1, respectively.

### RNA Synthesis and Oocyte Expression

Plasmid expression vectors were linearized by restriction enzyme digestion at sites downstream from the inserted cDNA. Linearized plasmids were purified with QIAQuik PCR purification kit (Qiagen), and transcription of cRNA was carried out using mMESSAGE mMACHINE SP6 kit (Ambion, Life Technologies). To achieve heterologous expression of human α4β2 nAChRs in two distinct subunit stoichiometries [(α4)_2_(β2)_3_ and (α4)_3_(β2)_2_], a well-established protocol was employed in which *Xenopus laevis* oocytes were injected with α4 and β2 cRNA in ratios of 1:10 and 10:1, respectively.^19,20^ A similar approach was employed to generate two stoichiometries of nAChRs containing mutated subunits (α4^L283A^, α4^S284A^, or β2^V278A^). Oocytes were injected, using a Drummond variable volume microinjector, with 32.2 nL of cRNA containing either a mixture of 30 ng/μL human α4 and 300 ng/μL human β2 or 300 ng/μL human α4 and 30 ng/μL human β2 cRNAs.

### Oocyte Electrophysiology

Adult female *Xenopus laevis* frogs were obtained from the European *Xenopus* Resource Centre at the University of Portsmouth. Animals were sacrificed using Schedule 1 procedures approved by the Animals (Scientific Procedures) Act 1986 and by the UCL Research Ethics Committee. *Xenopus* were anesthetized by immersion in 0.2% MS222 for 15 min (or until complete anesthesia was confirmed by absence of leg-withdrawal and righting reflex), followed by cranial concussion, decapitation, and pithing. *Xenopus* oocytes were isolated, maintained, and injected with cRNA, as described previously.^21^ Two-electrode voltage-clamp recordings were performed using a Warner Instruments OC-725C amplifier (Harvard Apparatus) with the oocyte membrane potential held at −60 mV, as described previously.^22^ Oocytes were continuously perfused with a modified Ringer’s solution (115 mM NaCl, 2.5 mM KCl, 1.8 mM BaCl_2_, and 10 mM HEPES, pH 7.3). Application of compounds was controlled by LabChart software (AD Instruments) using a BPS-8 solenoid valve solution exchange system (ALA Scientific Inc.). Typically, agonists were applied for 5 s or until a plateau in the response was observed. Antagonists were preapplied for 30 s and then coapplied with agonist for 5 s or until a plateau in the response was observed. Where data has been normalized to a maximum ACh response, the maximum response was determined from a minimum of three independent ACh dose–response curves.

### Statistical Analysis

For individual pairwise comparisons, statistical significance was determined using unpaired Student’s *t* tests or ANOVA for multiple comparisons. Dose–response curves were fitted by GraphPad Prism, using the following equation (where *I* is the current, *I*_max_ is the maximum current, the EC_50_ is the concentration of agonist that elicits a half-maximal response, and *n*_H_ is the Hill coefficient):
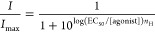


### Small Molecule Docking

To identify potential binding sites for quinolone antibiotics in the human (α4)_2_(β2)_3_ and (α4)_3_(β2)_2_ nAChRs, computational docking was performed with protein structures that have been determined previously by cryoelectron microscopy (Protein Data Bank codes 6CNJ and 6CNK, respectively).^23^ Small molecule computer docking was performed using AutoDock Vina (Molecular Graphics Lab at Scripps Research Institute, La Jolla, CA) and PLANTS (Protein–Ligand ANT System; Universität Tübingen, Germany). Docking was performed within a search area of 18 Å radius centered on the γ-carbon of T286 (α4) or T277 (β2) of the subunit corresponding to the principal (+) side of the subunit interface. This covered the inter- and intrasubunit cavities of the β2/α4 and α4/α4 interfaces of (α4)_3_(β2)_2_ (PDB code 6CNK) and the β2/α4 and β2/β2 interfaces of (α4)_2_(β2)_3_ (PDB code 6CNJ). With both docking programs, ligands were allowed to be fully flexible and the maximum search efficiency was used. One-thousand protein–ligand conformations were produced by each docking program for each interface query and analyzed with a previously described consensus docking protocol.^17^ This in-house script allows for a consensus binding mode or cluster to be identified from the protein–ligand conformations produced from the two independent docking programs. The rationale for this approach is to identify predicted binding sites for which there is a consensus between two docking programs that employ different scoring functions. The most highly populated consensus cluster of solutions (determined by RMSD with a cutoff of 2 Å between the two docking programmes) and highest ranked (by either PLANTS or AutoDock Vina scoring function) was taken to represent the active conformation of the ligand in each receptor stoichiometry.

## Results

### Antagonist Effects of Quinolone Antibiotics on (α4)_2_(β2)_3_ and (α4)_3_(β2)_2_ nAChRs

The effects of nine quinolone antibiotics (Figure 1) were examined by two-electrode voltage-clamp recording on heteromeric α4β2 nAChRs expressed in *Xenopus* oocytes. As has been reported previously, α4β2 nAChRs assemble into two subunit stoichiometries ((α4)_2_(β2)_3_ and (α4)_3_(β2)_2_) and these two distinct receptor populations can be generated in *Xenopus* oocytes by injection of differing ratios of α4 and β2 subunit cRNAs. In agreement with previous studies,^3,4,20^ oocytes expressing (α4)_2_(β2)_3_ nAChRs (injected with α4 and β2 cRNAs in a ratio of 1:10) were activated by ACh with an EC_50_ value of 1.6 ± 0.1 μM (*n* = 5), whereas oocytes expressing (α4)_3_(β2)_2_ nAChRs (injected with α4 and β2 cRNAs in a ratio of 10:1) were activated by ACh with an EC_50_ value of 37.1 ± 6.8 μM (*n* = 3).

**Figure 2:**
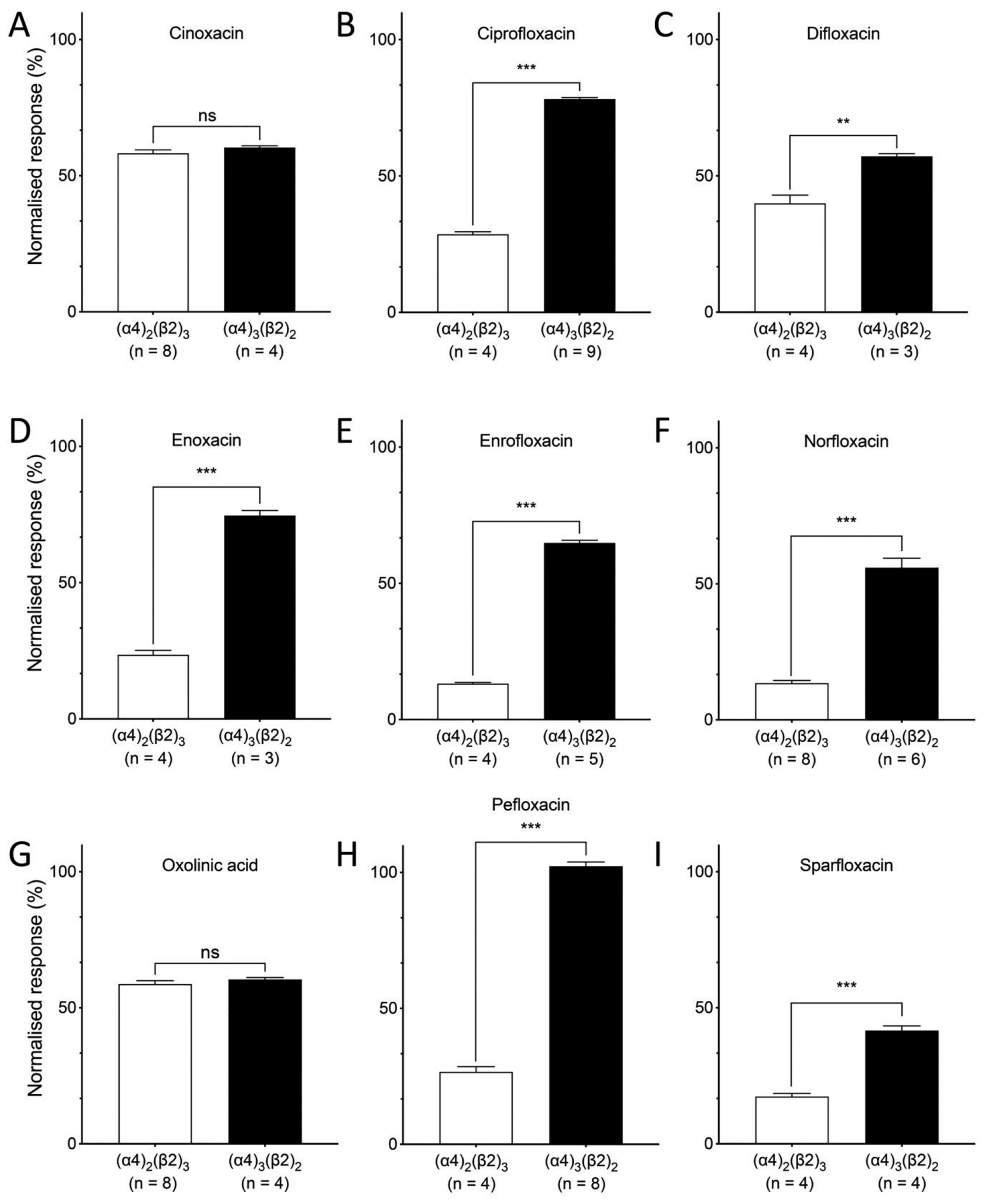
Inhibitory effects of quinolone antibiotics on α4β2 nAChRs: bar charts illustrating the effects of quinolone antibiotics on (α4)_2_(β2)_3_ nAChRs (white bars) and (α4)_3_(β2)_2_ nAChRs (black bars) expressed in *Xenopus* oocytes. Antibiotics (100 μM) were preapplied for 30 s and then coapplied with agonist (an EC_50_ concentration of ACh) for 5 s or until a plateau in the response. Responses are normalized to responses to ACh in the absence of antibiotic. Data are the mean ± SEM from at least three individual experiments (as indicated). Significant differences are indicated (∗∗ = *P* < 0.01, ∗∗∗ = *P* < 0.001, ns = not significant).

When applied alone to heterologously expressed α4β2 nAChRs, none of the quinolone antibiotics had any significant effect. However, when preapplied and coapplied with ACh, all displayed significant antagonist effects on at least one stoichiometry of α4β2 nAChRs (Figure 2 and Table 1). In initial studies, 100 μM of each antibiotic was coapplied with an EC_50_ concentration of ACh (1 μM ACh for (α4)_2_(β2)_3_ and 40 μM ACh for (α4)_2_(β2)_3_). Of the nine quinolone antibiotics examined, seven were fluoroquinolones (ciprofloxacin, difloxacin, enoxacin, enrofloxacin, norfloxacin, pefloxacin, and sparfloxacin) and all of these fluoroquinolone compounds displayed significantly greater antagonism on (α4)_2_(β2)_3_ nAChRs than on (α4)_3_(β2)_2_ nAChRs (Figure 2). This stoichiometry-selective antagonism was most apparent for pefloxacin, which inhibited responses to ACh on (α4)_2_(β2)_3_ nAChRs by 73.3 ± 1.9% (*n* = 4), whereas responses to ACh on (α4)_3_(β2)_2_ nAChRs were not significantly different in the presence or absence of pefloxacin (Figure 2H and Table 1). In addition, two nonfluorinated quinolone antibiotics were examined (cinoxacin and oxolinic acid). Once again, significant antagonist effects were observed, but in contrast to the fluoroquinolone antibiotics, there was no significant difference in the level of antagonism observed with (α4)_2_(β2)_3_ and (α4)_3_(β2)_2_ nAChRs (Figure 2A,G). In summary, stoichiometry-selective antagonism was displayed by all seven fluoroquinolone antibiotics examined, whereas nonselective antagonism was observed with both of the nonfluorinated quinolone antibiotics.

**Table 1:** Inhibitory Effects of Quinolone Antibiotics on (α4)_2_(β2)_3_ and (α4)_3_(β2)_2_ nAChRs^a^

antibiotic	(α4)_2_(β2)_3_ nAChR (% control response to ACh)	(α4)_3_(β2)_2_ nAChR (% control response ACh)
cinoxacin	58.2 ± 1.20 (*n* = 8)***	60.4 ± 0.61 (*n* = 4)***
ciprofloxacin	28.6 ± 0.99 (*n* = 4)***	78.2 ± 0.53 (*n* = 9)***
enoxacin	23.5 ± 1.65 (*n* = 4)***	74.6 ± 1.88 (*n* = 3)***
enrofloxacin	13.2 ± 0.46 (*n* = 4)***	64.8 ± 1.01 (*n* = 5)***
difloxacin	39.9 ± 3.02 (*n* = 4)***	57.2 ± 0.97 (*n* = 3)***
norfloxacin	13.0 ± 1.0 (*n* = 8)***	54.1 ± 3.40 (*n* = 6)***
oxolinic acid	59.5 ± 0.99 (*n* = 6)***	60.4 ± 0.72 (*n* = 4)***
pefloxacin	26.7 ± 1.9 (*n* = 4)***	104.1 ± 1.6 (*n* = 8)^NS^
sparfloxacin	17.4 ± 1.12 (*n* = 4)***	58.4 ± 1.72 (*n* = 8)***

a In all cases, inhibition was examined with 100 μM antibiotic coapplied with an EC_50_ concentration of ACh (1 μM for (α4)_2_(β2)_3_ and 40 μM for (α4)_3_(β2)_2_). Data are the mean ± SEM. Significant differences from control responses in the absence of antibiotic are indicated (***P < 0.001; NS = not significant).

Following our initial studies with a range of quinolone antibiotics, two compounds were selected for more detailed studies. These were pefloxacin, which displayed selective antagonism of (α4)_2_(β2)_3_ nAChRs, and cinoxacin, which displayed nonselective antagonism on (α4)_2_(β2)_3_ and (α4)_3_(β2)_2_ nAChRs.

### Antagonism of α4β2 nAChRs by Pefloxacin

**Figure 3:**
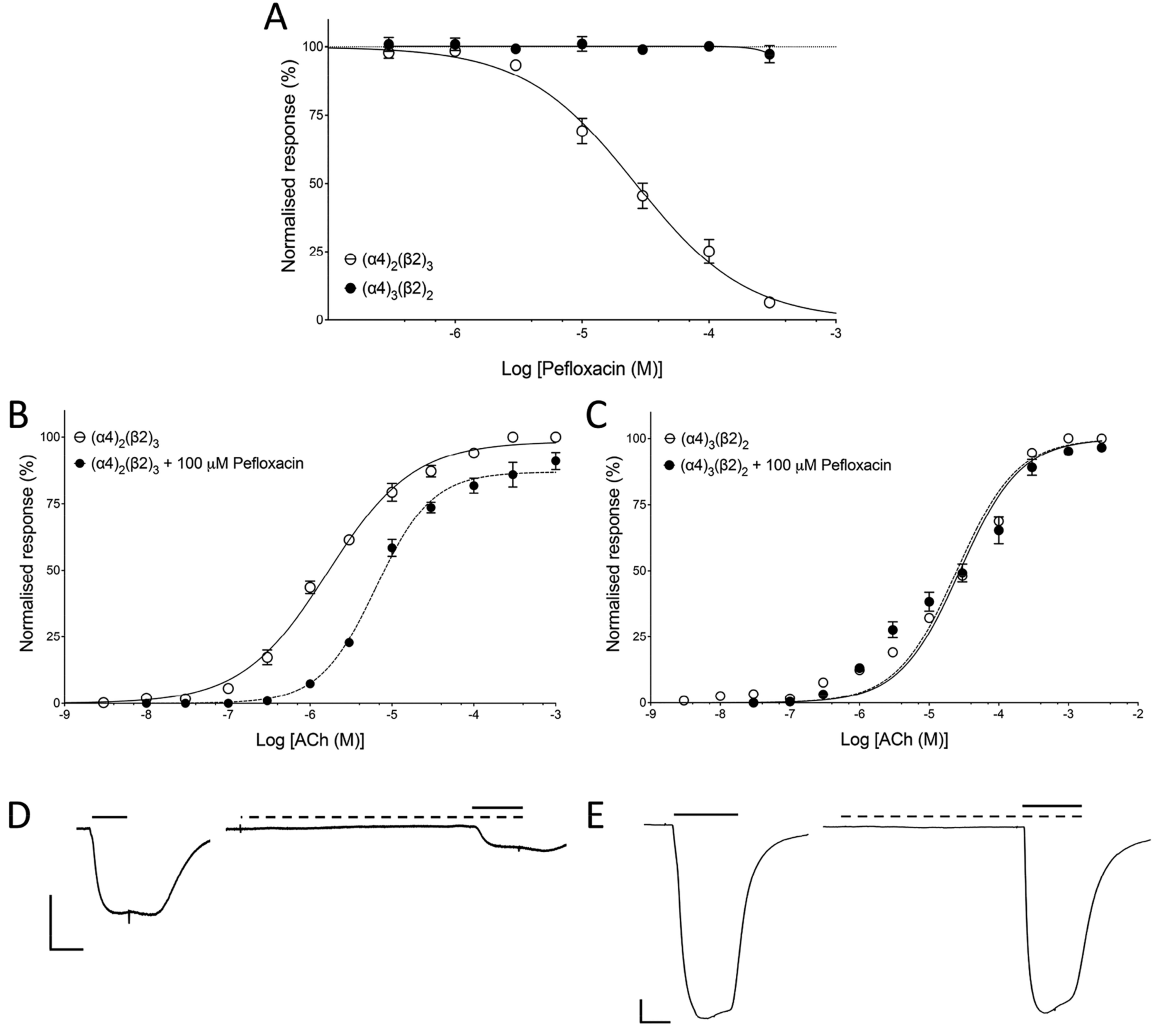
Effects of pefloxacin on (α4)_2_(β2)_3_ and (α4)_3_(β2)_2_ nAChRs expressed in *Xenopus* oocytes. (A) Effects of a range of concentrations of pefloxacin, preapplied and coapplied with an EC_50_ concentration of ACh on (α4)_2_(β2)_3_ (open circles) and (α4)_3_(β2)_2_ (filled circles). Data are the mean ± SEM of a least three experiments. (B) ACh dose–response curve with (α4)_2_(β2)_3_ nAChRs in the absence (open circles) and presence (filled circles) of pefloxacin (100 μM). Data are the mean ± SEM of a least three experiments. (C) ACh dose–response curve with (α4)_3_(β2)_2_ nAChRs in the absence (open circles) and presence (filled circles) of pefloxacin (100 μM). Data are the mean ± SEM of a least three independent experiments. (D) Representative traces from (α4)_2_(β2)_3_ nAChRs showing responses to an EC_50_ concentration of ACh in the absence (left) and presence (right) of pefloxacin (100 μM). Scale bars: 500 nA (vertical) and 5 s (horizontal). (E) Representative traces from (α4)_3_(β2)_2_ nAChRs showing responses to an EC_50_ concentration of ACh in the absence (left) and presence (right) of pefloxacin (100 μM). Scale bars: 500 nA (vertical) and 5 s (horizontal).

Oocytes expressing either (α4)_2_(β2)_3_ or (α4)_2_(β2)_3_ nAChRs were examined by coapplying a range of concentrations of pefloxacin with an EC_50_ concentration of ACh. With (α4)_3_(β2)_2_ nAChRs, pefloxacin showed no significant effect on responses to EC_50_ concentrations of ACh (Figure 3A). In contrast, with (α4)_2_(β2)_3_ nAChRs, pefloxacin inhibited responses with an IC_50_ value of 26.4 ± 3.4 μM, *n* = 4 (Figure 3A). When a fixed concentration of pefloxacin (100 μM) was coapplied with a range of ACh concentrations to (α4)_2_(β2)_3_ nAChRs, it caused a rightward shift of the ACh dose–response curve, together with a reduced maximal response to ACh (Figure 3B). Pefloxacin (100 μM) caused a significant shift in the ACh EC_50_ from 1.6 ± 0.1 μM (*n* = 5) to 6.4 ± 0.7 μM (*n* = 4) (*P* < 0.001) and reduced the maximal normalized ACh response to 91.0 ± 1.3% (*n* = 4; *P* < 0.001). Pefloxacin also caused a significant change (*P* < 0.0001) in the Hill coefficient from 0.83 ± 0.2 (*n* = 5) to 1.3 ± 0.1 (*n* = 4) (Figure 3B). In contrast, with (α4)_3_(β2)_2_ nAChRs, pefloxacin had no significant effect on responses to ACh, causing no changes in maximal response, EC_50_, or Hill coefficient (Figure 3B). This is consistent with pefloxacin acting as a selective noncompetitive antagonist of (α4)_2_(β2)_3_ nAChRs. Representative traces of ACh responses in the absence and presence of pefloxacin are shown (Figure 3D,E).

### Antagonism of α4β2 nAChRs by Cinoxacin

**Figure 4:**
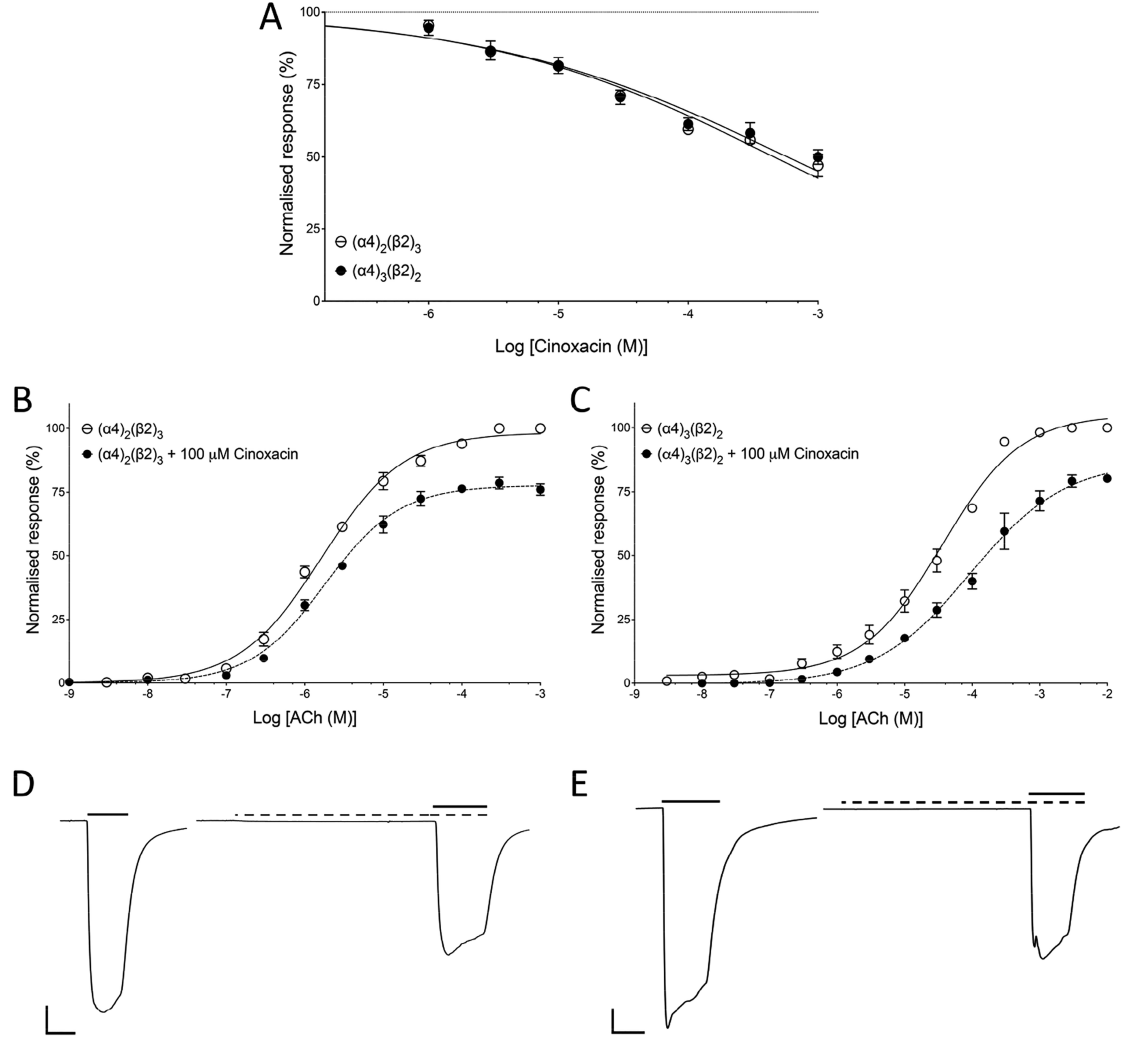
Effects of cinoxacin on human (α4)_2_(β2)_3_ and (α4)_3_(β2)_2_ nAChRs expressed in *Xenopus* oocytes. (A) Effects of a range of concentrations of cinoxacin, preapplied and coapplied with an EC_50_ concentration of ACh on (α4)_2_(β2)_3_ (open circles) and (α4)_3_(β2)_2_ (filled circles). Data are the mean ± SEM of a least three experiments. (B) ACh dose–response curve with (α4)_2_(β2)_3_ nAChRs in the absence (open circles) and presence (filled circles) of cinoxacin (100 μM). Data are the mean ± SEM of a least three experiments. (C) ACh dose–response curve with (α4)_3_(β2)_2_ nAChRs in the absence (open circles) and presence (filled circles) of cinoxacin (100 μM). Data are the mean ± SEM of a least three independent experiments. (D) Representative traces from (α4)_2_(β2)_3_ nAChRs showing responses to an EC_50_ concentration of ACh in the absence (left) and presence (right) of cinoxacin (100 μM). Scale bars: 500 nA (vertical) and 5 s (horizontal). (E) Representative traces from (α4)_3_(β2)_2_ nAChRs showing responses to an EC_50_ concentration of ACh in the absence (left) and presence (right) of cinoxacin (100 μM). Scale bars: 500 nA (vertical) and 5 s (horizontal).

A similar series of experiments were performed with cinoxacin. Oocytes expressing either (α4)_2_(β2)_3_ or (α4)_3_(β2)_2_ nAChRs were examined by coapplying a range of concentrations of the cinoxacin with an EC_50_ concentration of ACh. The level of antagonism observed with cinoxacin was similar on the two receptor populations. Cinoxacin (1 mM) inhibited (α4)_2_(β2)_3_ by 50.5 ± 4.5% (*n* = 4) and (α4)_2_(β2)_3_ by 50.0 ± 2.5% (*n* = 4) (Figure 4A). When a fixed concentration of cinoxacin (100 μM) was coapplied with a range of ACh concentrations, it resulted in an insurmountable antagonism of ACh responses with both (α4)_2_(β2)_3_ and (α4)_3_(β2)_2_ nAChRs (Figure 4B,C). Cinoxacin (100 μM) caused a significant shift in the ACh EC_50_ on (α4)_2_(β2)_3_ nAChRs from 1.6 ± 0.1 μM (*n* = 5) to 2.1 ± 0.1 μM (*n* = 4) (*P* = 0.01) and reduced the maximal normalized ACh response to 76.0 ± 1.5% (*n* = 4; *P* < 0.001). In addition, cinoxacin caused a significant change in the Hill coefficient from 0.83 ± 0.07 (*n* = 5) to 1.0 ± 0.1 (*n* = 4; *P* = 0.01) (Figure 4B). Cinoxacin (100 μM) also caused a significant shift in the ACh EC_50_ on (α4)_3_(β2)_2_ nAChRs from 37.1 ± 6.8 μM (*n* = 3) to 94.5 ± 3.1 μM (*n* = 4; *P* = 0.01) and reduced the maximal normalized ACh response to 80.0 ± 0.9% (*n* = 4; *P* < 0.001). There was also a significant change in the Hill coefficient from 0.74 ± 0. (*n* = 3) to 0.62 ± 0.2 (*n* = 4; *P* < 0.001) (Figure 4B). These findings are consistent with cinoxacin acting as a nonselective, noncompetitive antagonist of both (α4)_2_(β2)_3_ and (α4)_3_(β2)_2_ nAChRs. Representative traces of ACh responses in the absence and presence of cinoxacin are shown (Figure 4D,E).

### Docking of Quinolone Antibiotics into α4β2 nAChR Structures

**Figure 5:**
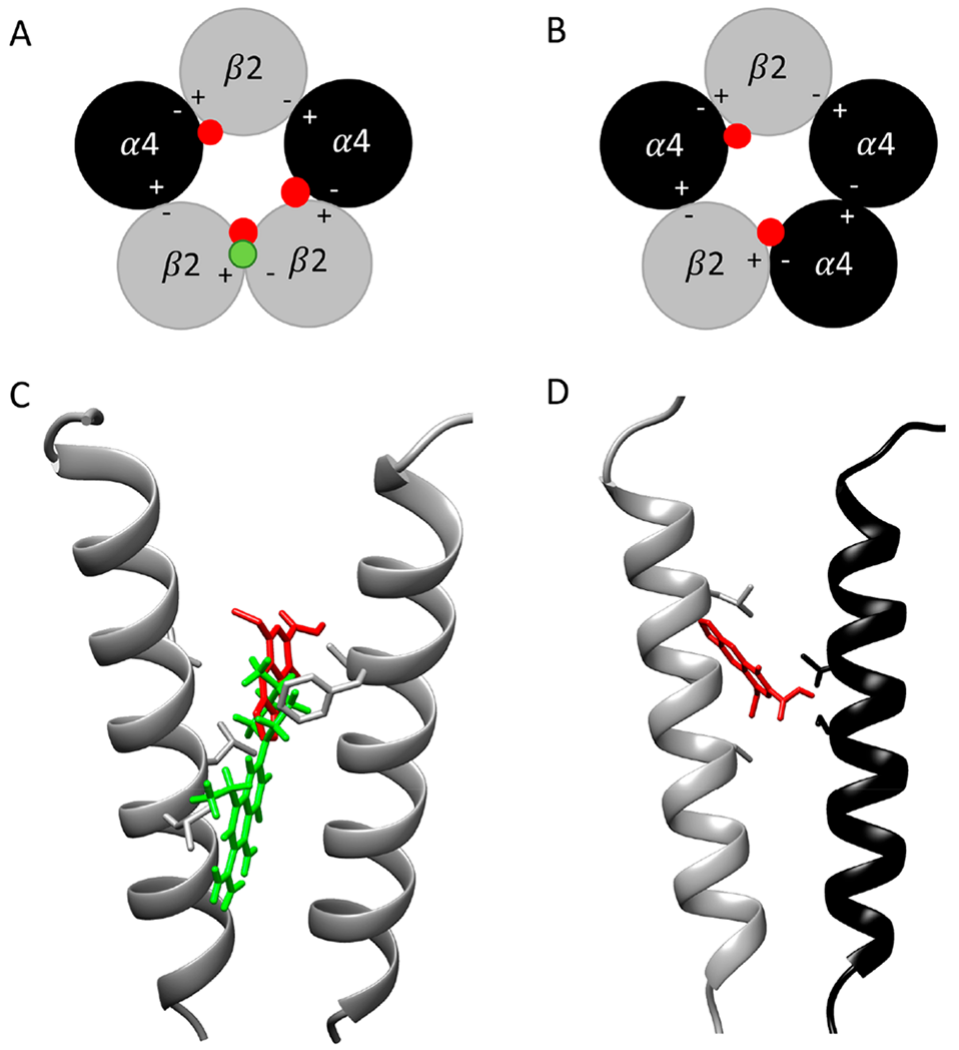
Computational docking of pefloxacin and cinoxacin into human (α4)_2_(β2)_3_ (PDB code 6CNJ) and (α4)_3_(β2)_2_ nAChRs structures (PDB code 6CNK). (A) Schematic representation of the docking sites of cinoxacin identified in the (α4)_2_(β2)_3_ nAChRs structures (red dots) and one site for pefloxacin (green dot). (B) Schematic representation of the docking sites of cinoxacin identified in the (α4)_3_(β2)_2_ nAChRs structures (red dots). (C, D) Docked positions of cinoxacin (red) and pefloxacin (green) in the transmembrane regions of (α4)_2_(β2)_3_ and (α4)_3_(β2)_2_, respectively. The images show the pore-lining TM2 transmembrane region of the β2(+)/β2(−) interface of (α4)_2_(β2)_3_ (C) and the β2(+)/α4(−) interface of (α4)_3_(β2)_2_ (D).

Computational docking studies were performed with three-dimensional atomic models of the (α4)_2_(β2)_3_ and (α4)_3_(β2)_2_ nAChRs that had been determined previously by cryoelectron microscopy (PDB codes 6CNJ and 6CNK, respectively).^23^ A consensus docking approach^17^ was employed, involving two independent docking methods (AutoDock Vina and PLANTS). Since previous studies had identified the intersubunit transmembrane region as being the most plausible binding site for allosteric modulators such as pefloxacin in the α7 nAChR,^16,17^ docking studies were performed within a search area of 18 Å radius centered in this region (see Materials and Methods). When results were compared from the two computational docking studies, no consensus binding site for pefloxacin was identified in the (α4)_3_(β2)_2_ nAChR subtype, whereas a single plausible consensus binding site was identified in (α4)_2_(β2)_3_ at the β2/β2 interface (Figure 5) at a location similar to that identified previously for allosteric modulators of nAChRs.^16,17^ These findings are consistent with evidence that pefloxacin is a selective antagonist of the (α4)_2_(β2)_3_ nAChR subtype. In contrast, docking studies with cinoxacin identified plausible binding sites in both receptor structures. Again, this is consistent with the finding that these compounds display no selectivity in their antagonist effects on (α4)_2_(β2)_3_ and (α4)_3_(β2)_2_ nAChRs. Three binding sites were identified within (α4)_2_(β2)_3_ (one within the β2/β2 interface and two within the β2/α4 interface) (Figure 5), and two binding sites were identified in the (α4)_3_(β2)_2_ nAChR (both within the β2/α4 interface) (Figure 5).

Further docking studies were performed with the other seven quinolone antibiotics that had been examined on nAChRs expressed in *Xenopus* oocytes (ciprofloxacin, difloxacin, enoxacin, enrofloxacin, norfloxacin, oxolinic acid, and sparfloxacin). These are compounds that, like cinoxacin, displayed antagonist effects on both α4β2 stoichiometries. As was observed with docking studies with cinoxacin (but in contrast to pefloxacin), plausible binding sites were identified for all seven of these compounds in both α4β2 stoichiometries and in positions that closely resembled those that had been identified with cinoxacin.

### Effects of Pefloxacin and Cinoxacin on Mutant α4β2 nAChRs

A possible explanation for the nonselective antagonism by compounds such as cinoxacin and for the selective antagonism by pefloxacin might be that cinoxacin is able to bind to subunit interfaces containing the α4 subunit, whereas pefloxacin binds selectively at the interface of two β2 subunits. Such an explanation would also be consistent with the computer docking studies. With the aim of testing this hypothesis, the influence of α4 subunit mutations was examined on the antagonist effects of pefloxacin and cinoxacin. Two amino acids within the transmembrane domain of the α4 subunit were selected for site-directed mutagenesis (L283 and S284) due to their close proximity to the predicted binding sites of cinoxacin and the lack of proximity to the predicted binding site for pefloxacin. A further reason for selecting these two amino acids was that mutagenesis of the analogous amino acids in α7 nAChRs has been shown to alter allosteric modulation by compounds such as pefloxacin.^16^ Both amino acids were mutated individually to alanine to create α4^L283A^ and α4^S284A^. In addition, an amino acid within the transmembrane domain of the β2 subunit (V278) was selected for site-directed mutagenesis due to its proximity to the predicted binding site of both cinoxacin and pefloxacin and was mutated to alanine to create β2^V278A^.

**Figure 6:**
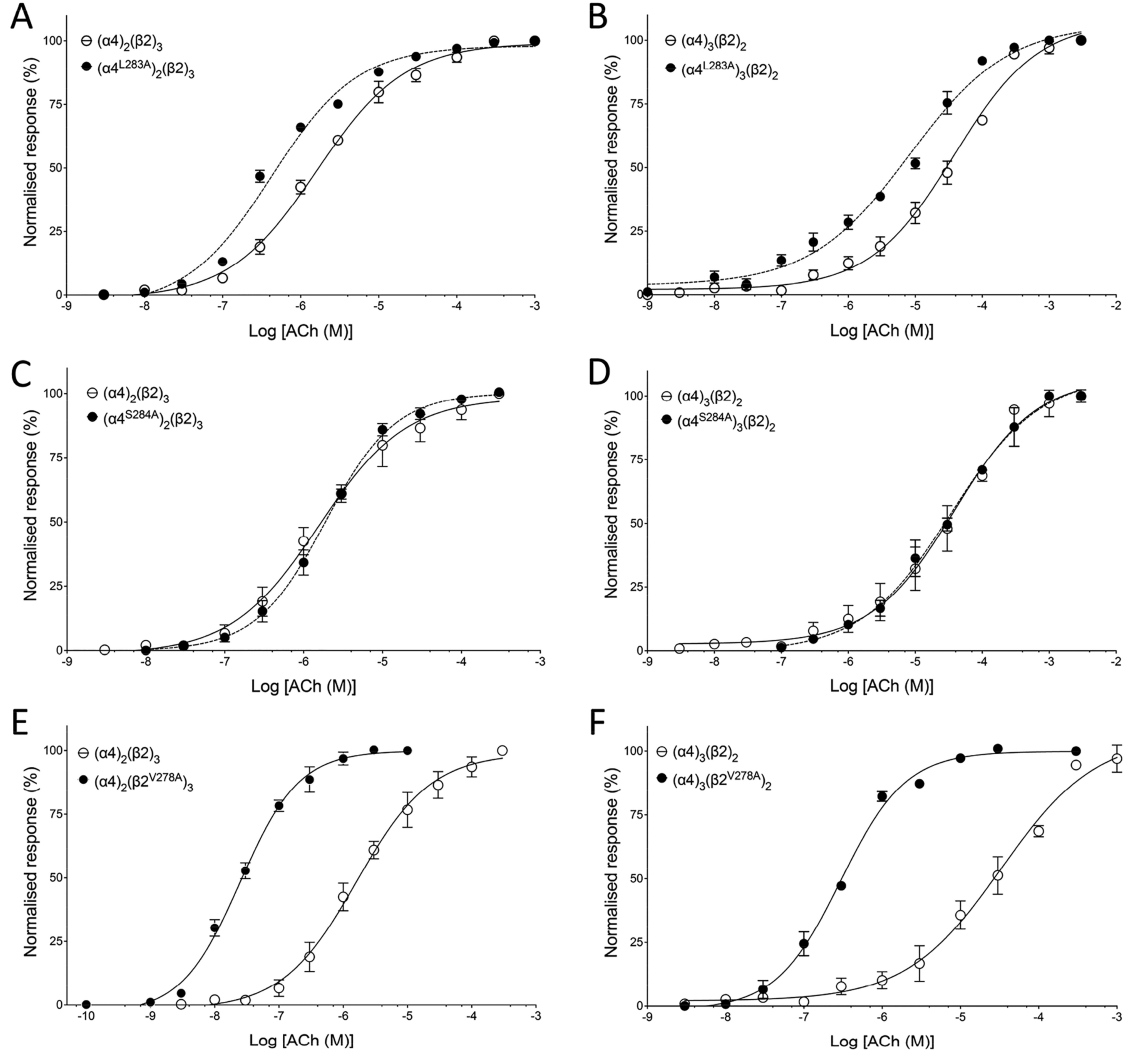
Agonist (ACh) sensitivity of α4β2 nAChRs containing α4^L283A^, α4^S284A^, or β2^V278A^ mutations. ACh concentration–response curve for (A) (α4)_2_(β2)_3_ (open circles) and (α4^L283A^)_2_(β2)_3_ (filled circles), (B) (α4)_3_(β2)_2_ (open circles) and (α4^L283A^)_3_(β2)_2_ (filled circles), (C) (α4)_2_(β2)_3_ (open circles) and (α4^S284A^)_2_(β2)_3_ (closed circles), (D) (α4)_3_(β2)_2_ (open circles) and (α4^S284A^)_3_(β2)_2_ (filled circles), (E) (α4)_2_(β2)_3_ (open circles) and (α4)_2_(β2^V278A^)_3_ (filled circles), and (F) (α4)_3_(β2)_2_ (open circles) and (α4)_3_(β2^V278A^)_2_ (filled circles). All data are normalized to the maximum ACh response and are the mean ± SEM of at least three independent experiments.

Receptors containing transmembrane mutations were generated by injecting cRNA encoding α4^L283A^, α4^S284A^, or β2^V278A^ along with wild-type subunit cRNA in the ratio 1:10 or 10:1, and dose–response curves to ACh were generated (Figure 6). The α4^S284A^ mutation had no significant effect on the EC_50_ value for ACh compared with that of wild-type α4β2 (Figure 6C,D), but the α4^L283A^ and β2^V278A^ mutations caused a significant leftward shift in the ACh concentration–response curve for both stoichiometries (Figure 6). Receptors containing α4^L283A^ with the assumed stoichiometry of (α4^L283A^)_2_(β2)_3_ had an ACh EC_50_ of 422.3 ± 42.9 nM (*n* = 3), which is significantly different (*P* = 0.0009) from that of the wild-type (α4)_2_(β2)_3_ nAChR. In addition, those with the assumed stoichiometry of (α4^L283A^)_3_(β2)_2_ had an ACh EC_50_ of 11.42 ± 3.5 μM (*n* = 3), which is significantly different (*P* = 0.029) from that of the corresponding wild-type nAChR. Similarly, receptors containing β2^V278A^ with an assumed stoichiometry of (α4)_2_(β2^V278A^)_3_ had an ACh EC_50_ of 24.9 ± 4.1 nM (*n* = 3), which is significantly different (*P* < 0.001) from that of the wild-type (α4)_2_(β2)_3_ nAChR. In addition, those with an assumed stoichiometry of (α4)_3_(β2^V278A^)_2_ had an ACh EC_50_ value of 294.8 ± 34.3 nM (*n* = 3), which is significantly different (*P* < 0.001) from that of the corresponding wild-type nAChR.

**Figure 7:**
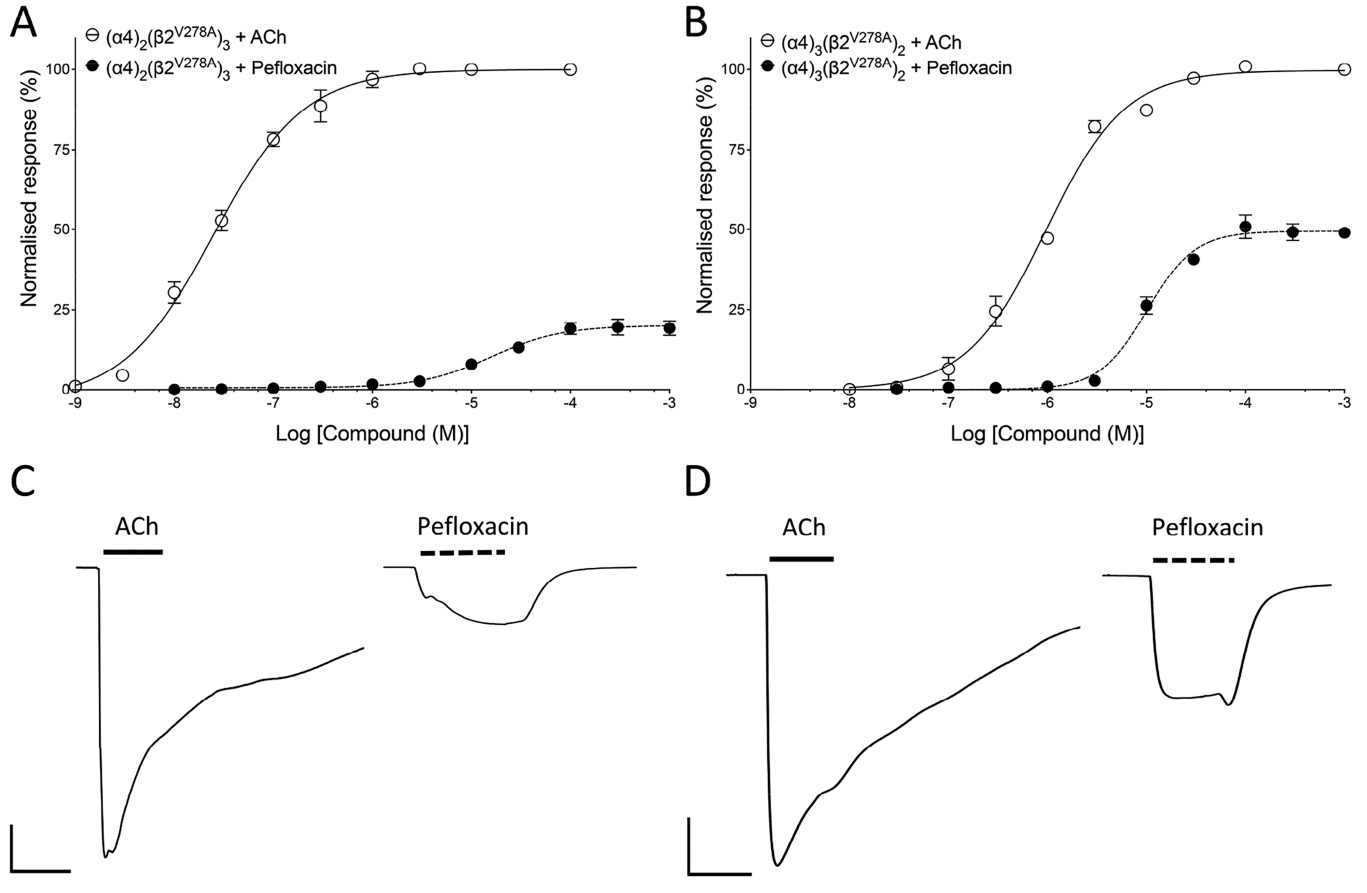
Agonist effects of pefloxacin on α4β2 nAChRs containing the β2^V278A^ mutation. (A) Agonist dose–response curves for ACh (open circles) and pefloxacin (filled circles) on (α4)_2_(β2^V278A^)_3_. Data are normalized to maximal ACh responses (100 μM) and are the mean ± SEM of at least three experiments. (B) Representative traces of maximal ACh response (100 μM) and maximal pefloxacin response (1 mM) of (α4)_2_(β2^V278A^)_3_. Scale bars: 500 nA (vertical) and 5 s (horizontal). (C) Agonist dose–response curves for ACh (open circles) and pefloxacin (filled circles) on (α4)_2_(β2^V278A^)_3_. Data are normalized to maximal ACh responses (1 mM) and are the mean ± SEM of at least three experiments. (D) Representative traces of maximal ACh response (1 mM) and maximal pefloxacin response (1 mM) of (α4)_3_(β2^V278A^)_2_. Scale bars: 500 nA (vertical) and 5 s (horizontal).

As was found with wild-type α4β2 nAChRs, cinoxacin had no effect when applied alone to α4β2 nAChRs containing mutated α4^L283A^, α4^S284A^, or β2^V278A^ subunits. Similarly, pefloxacin had no effect when applied alone to α4β2 nAChRs containing mutated α4^L283A^ or α4^S284A^ subunits. In contrast, when pefloxacin was applied alone to α4β2 nAChRs containing the β2^V278A^ subunit, weak agonist effects were observed (Figure 7). When applied alone to (α4)_2_(β2^V278A^)_3_, pefloxacin generated maximal normalized responses of 10.1 ± 1.1% (*n* = 4) with an EC_50_ of 15.9 ± 1.2 μM (*n* = 4) (Figure 7A,C). Similarly, on (α4)_3_(β2^V278A^)_2_ pefloxacin generated maximal normalized responses of 48.9 ± 1.8% (*n* = 4) with an EC_50_ of 15.9 ± 1.2 μM (*n* = 4) (Figure 7B,D). These findings indicate that, in contrast to the two transmembrane mutations examined on the α4 subunit, the β2^V278A^ transmembrane mutation converts pefloxacin (but not cinoxacin) into a partial agonist.

**Figure 8:**
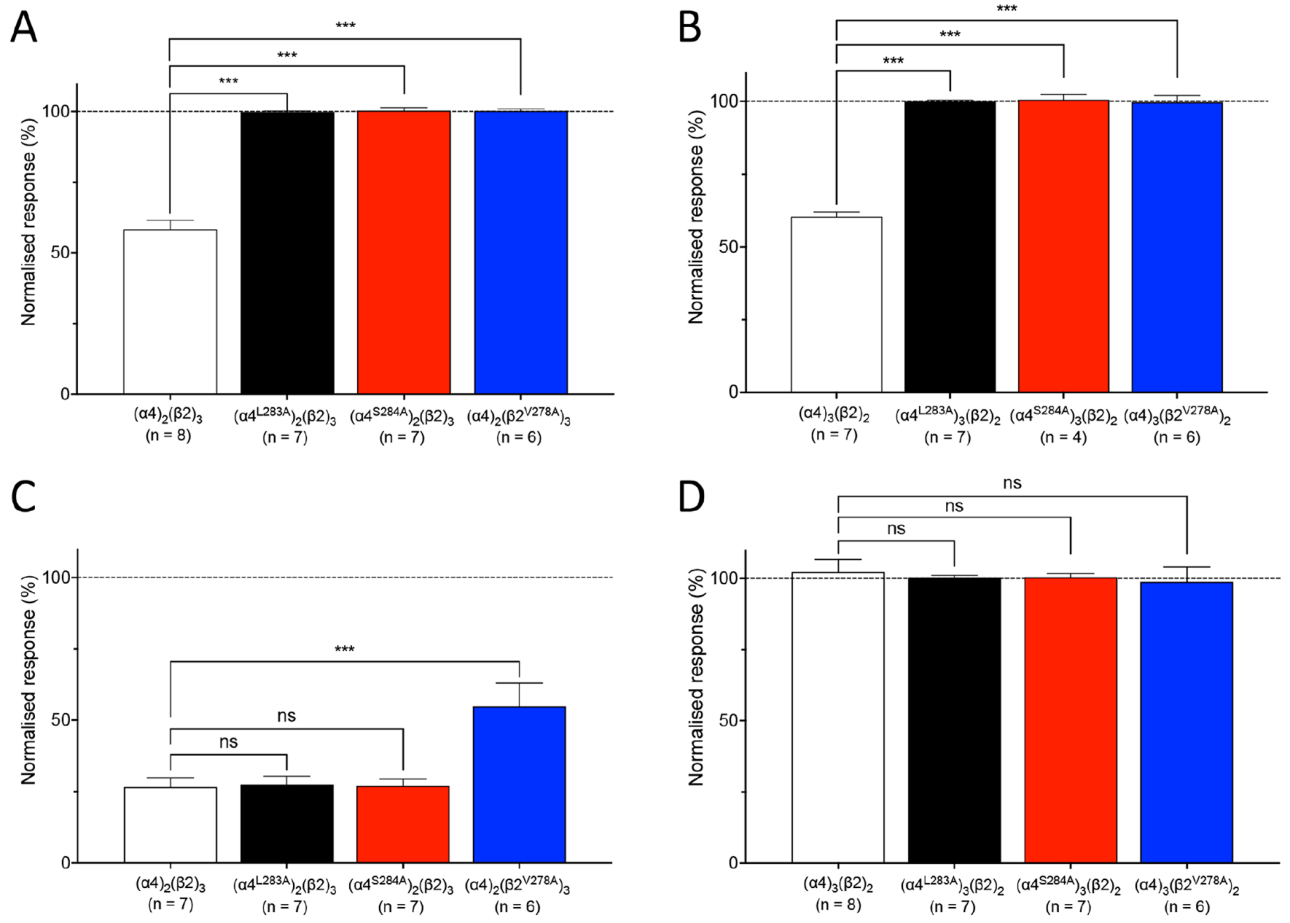
Influence of cinoxacin and pefloxacin of α4β2 nAChRs containing α4^L283A^, α4^S284A^, or β2^V278A^ mutations. Bar graphs illustrate the effects of 100 μM cinoxacin (A, B) and pefloxacin (C, D) on responses to an EC_50_ concentration of ACh. Data are presented for (α4)_2_(β2)_3_ (A, C; white bars), (α4^L283A^)_2_(β2)_3_ (A, C; black bars), (α4^S284A^)_2_(β2)_3_ (A, C; red bars), (α4)_2_(β2^V278A^)_3_ (A, C; blue bars), (α4)_3_(β2)_2_ (B, D; white bars), (α4^L283A^)_3_(β2)_2_ (B, D; black bars), (α4^S284A^)_3_(β2)_2_ (B, D; red bars), and (α4)_3_(β2^V278A^)_2_ (B, D; blue bars). All data are normalized to responses to an EC_50_ concentration of ACh and are the mean ± SEM of at least three independent experiments. Significant differences are indicated (∗∗∗ = *P* < 0.001, ns = not significant).

The effects of cinoxacin (100 μM) on responses to an EC_50_ concentration of ACh were examined in α4β2 nAChRs containing mutated α4^L283A^, α4^S284A^, or β2^V278A^ subunits in both stoichiometries. Each of the three transmembrane mutations abolished the antagonist effect of cinoxacin in both stoichiometries of α4β2 nAChRs (Figure 8A,B). In contrast, when pefloxacin was coapplied with ACh, neither of the α4 subunit mutations had a significant effect on the antagonist effect of pefloxacin (Figure 8C,D). For receptors containing a mutated α4 subunit in the stoichiometry (α4)_2_(β2)_3_, pefloxacin exhibited antagonist effects that were not significantly different from those observed with wild-type (α4)_2_(β2)_3_ nAChRs (Figure 7C). For receptors containing a mutated α4 subunit in the stoichiometry (α4)_3_(β2)_2_, pefloxacin exerted no significant antagonist effect (Figure 8D). Examining the inhibitory effect of pefloxacin on receptors containing the β2^V278A^ mutation may be harder to interpret due to this mutation converting pefloxacin into a partial agonist (as was described earlier). Nevertheless, the effects of pefloxacin (100 μM) on responses to an EC_50_ concentration of ACh were examined on α4β2 nAChRs containing the β2^V278A^ and were broadly similar to those observed with the α4 mutations (Figure 8C,D). As was the case with both wild-type (α4)_3_(β2)_2_ nAChRs and (α4)_3_(β2)_2_ nAChRs containing an α4 mutation, pefloxacin caused no significant inhibition of responses to ACh on (α4)_3_(β2^V278A^)_2_ (Figure 8D). Pefloxacin acted as an inhibitor of ACh responses on (α4)_2_(β2^V278A^)_3_ nAChRs, as it did with wild-type (α4)_2_(β2)_3_ nAChRs and (α4)_2_(β2)_3_ nAChRs containing an α4 mutation, but caused a significantly lower level of inhibition (*P* < 0.001) (Figure 8C). Therefore, all three transmembrane mutations produce effects that are consistent with the hypothesis that pefloxacin and cinoxacin modulate α4β2 nAChRs though different binding sites or mechanisms.

## Discussion

A notable aspect of the present study is that fluoroquinolone antibiotics exhibit stoichiometry-selective antagonism of α4β2 nAChRs. The effect was most pronounced for pefloxacin, which exhibits complete selectivity for α4β2 nAChRs in the (α4)_2_(β2)_3_ stoichiometry. The primary difference between the two α4β2 nAChR stoichiometries is the presence of a β2/β2 interface in (α4)_2_(β2)_3_ and an α4/α4 interface in the (α4)_3_(β2)_2_. It is of interest, therefore, that computational docking studies are consistent with the possibility that pefloxacin binds preferentially to a site at the β2/β2 interface in (α4)_2_(β2)_3_ nAChRs, whereas less selective and nonselective quinolone antibiotics were predicted to interact with sites at both the β2/β2 and β2/α4 subunit interfaces. Although plausible binding sites were identified in (α4)_2_(β2)_3_ for all nine quinolone antibiotics examined, the predicted binding site for pefloxacin is qualitatively distinct from that of the other compounds, extending deeper into the intersubunit cavity within the β2/β2 subunit interface. In addition, while cinoxacin and the other quinolone antibiotics are predicted to interact with TM2 of both subunits at the β2/β2 interface, pefloxacin is predicted to also interact with the TM1 and TM3 helices of the complementary and primary subunits, respectively. This supports the possibility that pefloxacin may make important interactions with the β2/β2 interface that are distinct from that of the other antibiotics examined.

A prediction, based on our docking results, was that mutations in the α4 subunit and close to the predicted intersubunit transmembrane binding site of quinolone antibiotics might have a more profound effect on nonselective antibiotics such as cinoxacin (that were predicted to bind at both the β2/α4 and β2/β2 subunit interfaces) than pefloxacin (that was predicted to bind exclusively at the β2/β2 subunit interface), having found that two such mutations (α4^L283A^ and α4^S284A^) abolish the antagonist effects of cinoxacin but have no significant effect on pefloxacin supports the predictions. These particular amino acids were selected for mutagenesis studies because they are at positions in the α4 subunit that are analogous to two amino acids in the α7 nAChR (α4^S248^ and α7^L247^) that have been shown previously to modulate the effects of compounds predicted to bind in the intersubunit transmembrane cavity.^16^ In addition, a mutation was made within the β2 subunit (V278A) at a site that is in close proximity to the predicted binding site of both cinoxacin and pefloxacin. As was seen with nAChRs containing α4 transmembrane mutations, the inhibitory effects of cinoxacin were abolished by this mutation in both stoichiometries. Interestingly, the β2^V278A^ mutation converted pefloxacin but not cinoxacin into a partial agonist. There are previous examples of transmembrane mutations converting antagonists into agonist, a finding that is probably a consequence of the mutations causing conformational changes that alter the energy barrier for transitions between open and closed states following ligand binding or by allowing bound ligands to more easily stabilize the open conformation. One of the best characterized examples is a transmembrane mutation in the nAChR α7 subunit (L247T) that causes increase spontaneous openings, reduces receptor desensitization, alters temperature sensitivity, and converts antagonists into agonists.^24−27^ Similarly, this α7 nAChR mutation can convert both positive allosteric modulators and silent allosteric modulators into allosteric agonists.^28−30^ In addition, several other nAChR transmembrane mutations have been reported that convert positive allosteric modulators into either agonists or antagonists.^17,31^

It is of interest that whereas some degree of selectivity for (α4)_3_(β2)_2_ nAChRs was observed with all fluoroquinolone antibiotics, no selectivity between (α4)_2_(β2)_3_ and (α4)_3_(β2)_2_ was seen with the two nonfluorinated quinolone antibiotics (cinoxacin and oxolinic acid). However, in contrast to the situation with pefloxacin, our docking studies do not provide a comprehensive explanation for this difference. Indeed, all of these antibiotics (with the exception of pefloxacin) were predicted to bind in broadly similar locations. However, it may be worth noting that cinoxacin and oxolinic acid were predicted to bind at positions in the transmembrane intersubunit cavity, while larger fluoroquinolones bound at position closer to the central ion channel and extended into the pore. Our findings extend previous evidence demonstrating that a variety of nicotinic ligands can show selectivity for the different stoichiometries of α4β2 nAChRs. This includes evidence for the stoichiometry-selective modulation of α4β2 nAChRs by agonists,^3,4,19,20,32−34^ competitive antagonists,^3,19^ divalent cations,^32,35^ and positive allosteric modulators.^36−42^

It has been estimated that between 1% and 4% of individuals treated with quinolone antibiotics display adverse side effects, including headaches, insomnia, and in some cases convulsions that become more prevalent when quinolone antibiotics are coadministered with nonsteroidal anti-inflammatory drugs.^43−45^ It has been suggested that these side effects are mediated via interactions with GABA_A_ receptors, since inhibitors of these receptors are proconvulsant, whereas potentiators are anxiolytic and sedative.^46^ Radioligand binding experiments have demonstrated that quinolone antibiotics can inhibit the binding of [^3^H]GABA or [^3^H]muscimol to GABA_A_ receptors in preparations of rat or mouse brain synaptic membranes. Furthermore, this inhibition was shown to be more potent when the antibiotics were coadministered with biphenylacetic acid, a nonsteroidal anti-inflammatory drug.^12,13^ Subsequently, whole-cell voltage-clamp recordings of rat dorsal root ganglion neurons and hippocampal neurons have demonstrated inhibition of GABA-evoked responses of GABA_A_ receptors by quinolone antibiotics, an effect that was also increased by the presence of biphenylacetic acid.^13,15,47^ In contrast, radioligand binding experiments have shown no effects of quinolone antibiotics on agonist binding to excitatory glutamate receptors, muscarinic acetylcholine receptors, and GABA_B_ receptors.^48,49^ It is unclear whether the antagonist effects of quinolone antibiotics observed on nAChRs have any relevance to the side effects that are sometimes reported, but it is of interest that they can exert significant effects on both inhibitory GABA_A_ receptors and excitatory nAChRs, both members of the superfamily of pentameric ligand-gated ion channels.

In previous studies, pefloxacin has been shown to be a noncompetitive antagonist of α7 nAChR and was originally identified on the basis of virtual screening for compounds predicted to interact with an allosteric transmembrane site on the α7 nAChR.^16^ Here we have obtained evidence of insurmountable antagonism with both pefloxacin and cinoxacin on α4β2 nAChRs that is consistent with them acting as noncompetitive antagonists of α4β2 nAChRs. It is well-known that the pharmacological properties of nAChRs are influenced by subunit composition, but the present study provides further evidence that such properties can also be influenced by the same subunits being arranged in different stoichiometries.
